# Treatment of genu valgum in children by means of temporary hemiepiphysiodesis using eight-plates: short-term findings

**DOI:** 10.1186/s12891-017-1823-7

**Published:** 2017-11-15

**Authors:** Dirk Zajonz, Eckehard Schumann, Magdalena Wojan, Fabian B. Kübler, Christoph Josten, Ulf Bühligen, Christoph E. Heyde

**Affiliations:** 1 Department of Orthopaedic Surgery, Trauma Surgery and Plastic Surgery, University Hospital Leipzig, Liebigstrasse 20, D-04103 Leipzig, Germany; 2 Department of Pediatric Surgery, University Hospital Leipzig, Liebigstrasse 20a, 04103 Leipzig, Germany

**Keywords:** Hemiepiphysiodesis, Eight-plate, Genu valgum, Growth modulation

## Abstract

**Background:**

Idiopathic genu valgum is a frequently diagnosed growth disorder in adolescence. Whenever the possibilities of conservative therapy have been exhausted, leg straightening by means of hemiepiphysiodesis has become the standard form of treatment. Because of their flexible screw-plate connection, eight-Plates have been reported in the literature to lead to lower complications regarding implant loosening and fracture compared to other implants.

The aim of this retrospective survey was to analyse our own patient population who were treated for genu valgum by means of temporary hemiepiphysiodesis near the knee using eight-Plates to modulate growth.

**Methods:**

Between July 2007 and July 2015, 198 eight-Plates were implanted near the knee in 132 children suffering from genu valgum to modulate growth. Depending on the deformity analysis, an eight-Plate was implanted on the distal medial femur and/or the proximal medial tibia. By December 2015, they had been removed from 105 patients. The etiology of genu valgum was mainly idiopathic or associated withobesity. Evaluation was carried out clinically and radiologically (whole-leg X-ray in standing) including determination of the joint angles.

**Results:**

The median follow-up period was about 46 months (12–102 months). The median age at implantation was 12.7 +/−6.76 years. Of the 105 patients, 45.7% (*n* = 48) were girls. The eight-Plates remained in place for a median period of 13 +/−1.76 months. Irrespective of the location of hemiepiphysiodesis, the intermalleolar distance was corrected to a median of 0 +/−2.1 cm while the anatomical femorotibial angle was corrected by on average 9 +/−2.7 °Mechanical lateral distal femoral angle changed an average 7 +/− 7.72 degrees. Medial proximal tibial angle changed an average 4 +/− 6.02 degrees. Complications necessitating surgery occurred in 2.8% of cases (1 wound infection, 3 corrective osteotomies following overcorrection).

**Conclusion:**

Temporary hemiepiphysiodesis using eight-Plates is a gentle, simple and effective procedure used to treat genu valgum by modulating growth. Slight overcorrection is desirable due to the rebound phenomenon, especially in young patients with high growth potential and risk groups such as obese children. In adolescents with only low growth potential (older than 14 years), owing to the low correction potential, the indication should be strictly reviewed and the possible failure of therapy should be discussed with the patient. No differences were observed regarding the location of the implanted eight-Plates (femoral or tibial).

## Background

Idiopathic genu valgum is a frequently diagnosed growth disorder in adolescence, albeit with strong variations regarding degree, cause and therapeutic relevance. Particularly in the growth phase from 2 to 11 years of age, a minor valgus deformity of 5–10° is regarded as physiological and may persist beyond this age interindividually and depending on constitution [1, 2]. These development-related changes in growth usually correct themselves spontaneously and in cases of mild persistent genu valgum can be successfully treated conservatively [1]. In the event of asymmetrical or unilateral deformities, metabolic, genetic, posttraumatic and other causes need to be excluded [3–5]. Given such causes, as with genu valgum due to obesity which is usually symmetrical, persistence or progress is to be expected. This has adverse effects on the further development and mobility of the affected child and may contribute to the early development of gonarthrosis [5–7]. Therefore, if the possibilities of conservative therapy have been exhausted, surgical leg straightening is the therapeutic standard.

Since the initial description by Blount and Clarke in 1949, hemiepiphysiodesis has become the gold standard, in particular as an alternative to osteotomy for leg straightening in adolescents [8]. Various techniques for epiphysiodesis involving screws, staples and wires have been trialled and described [9–14]. However, despite good success rates, complications such as material fractures and implant loosening as well as damage to growth plates have frequently been reported [9–11].

Eight-Plates (Orthofix, USA) first described by Stevens are simple to use [15]. Moreover, owing to the flexible screw/plate connection, fewer complications in terms of implant loosening and fracture can be expected. Good results as well as low rebound and complication rates have been recorded for the first series of cases [15–17].

The aim of this retrospective survey is to analyse our own patient population suffering from genu valgum who were treated with eight-Plates by means of temporary hemiepiphysiodesis near the knee to modulate growth. As well as the success rates, complications and rebound rates were to be determined, especially as a function of the plate positioning (tibial, femoral or femorotibial) and compared to recent literature.

## Methods

At the beginning of this study, approval was granted by Leipzig University’s ethics committee (432/16-ek). Parents or guardians gave their written consent in the treatment contract to the use of their children’s anonymized data.

Children with clinically conspicuous knock knees and an enlarged intermalleolar distance (> 10 cm) and with increasing valgus despite conservative therapy had their legs X-rayed anterior-posterior standing up. The determination of the intermalleolar distance to >10 cm is an internal clinical standard which has proved itself for years. It serves as an orientation and is shared with the increasing valgus deformity in conservative therapy.These X-rays were used to verify the valgus deformity and to exclude secondary causes. The anatomical femorotibial angle (aFTA), the anatomical lateral distal femoral angle (aLDFA: 79–83°) and the medial proximal tibial angle (MPTA: 85–90°) were measured in order to attribute the error to one or both joints. In the event of pathological angles, an assessment was carried out as to whether medial distal femoral and/or medial proximal tibial hemiepiphysiodesis should be carried out [1, 2].

**Fig. 1:**
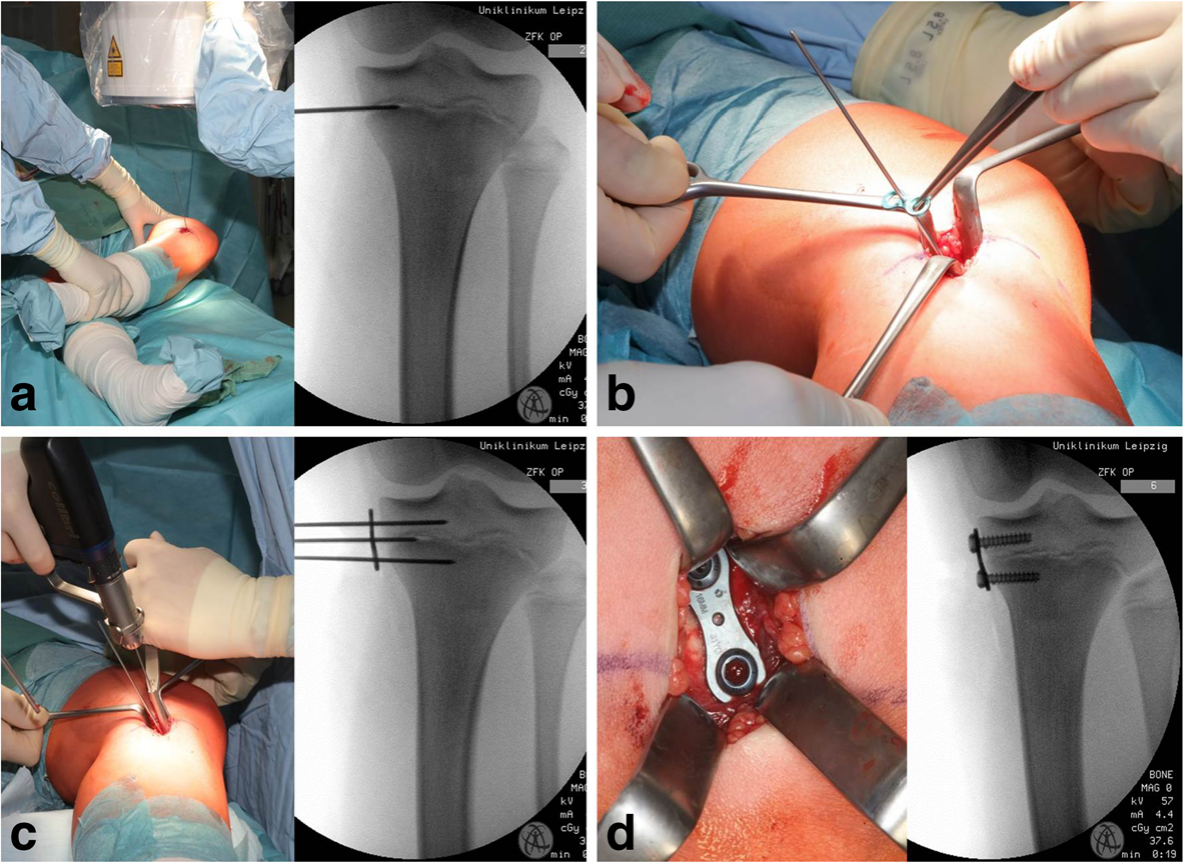
Surgical field during the implantation of a proximal medial tibial eight-Plate: **a:** marking of the growth plate using a K-wire with the aid of X-ray imaging; **b:** the eight-Plate implant is inserted through the corresponding hole and placed in the middle of the plate via the K-wire lying in the growth plate; **c:** the cancellous screws are then positioned using previously positioned K-wires; **d:** the inserted eight-Plate after implantation

The eight-Plate implant (Orthofix, Texas, USA) an approved medical product, was used. It consists of a plate shaped like a figure eight and two cannulated cancellous bone screws (Fig. 1) [18].

Surgery takes place using a standardized procedure after preparation under general anaesthetic. The positions of the growth plate (epiphyseal plate) and the implants are verified by the application of Kirschner’s wires (K-wires) with the aid of intraoperative X-ray imaging. The intraoperative procedure is shown in Fig. 1. (Fig. 1) Postoperatively, patients were mobilized under full weight-bearing with the knees allowed to move freely. Clinical follow-up took place every three months, which included measurement of the intermalleolar distance. In the event of clinically corrected genu valgum or slight overcorrection, an X-ray of the entire leg was taken to check the success of leg straightening. Following corrected leg axis, slight overcorrection, or the closure of the growth plates, the implants were removed to permit further growth. After removal, clinical follow-up examinations were carried out at first every six months in the first year and then annually.

Radiological findings were evaluated using SIENET MagicWeb (Siemens Medical Solutions, Erlangen, Germany). The angles were determined by means of the integrated, certified software. Statistical analysis was carried out using the spreadsheet software Microsoft Excel (Microsoft Corporation, Redmond, USA). For significance calculation, the two-sample t-test was used for two independent samples. The significance level α was set at 5% (α = 0.05 (5%)).

## Results

**Fig. 2:**
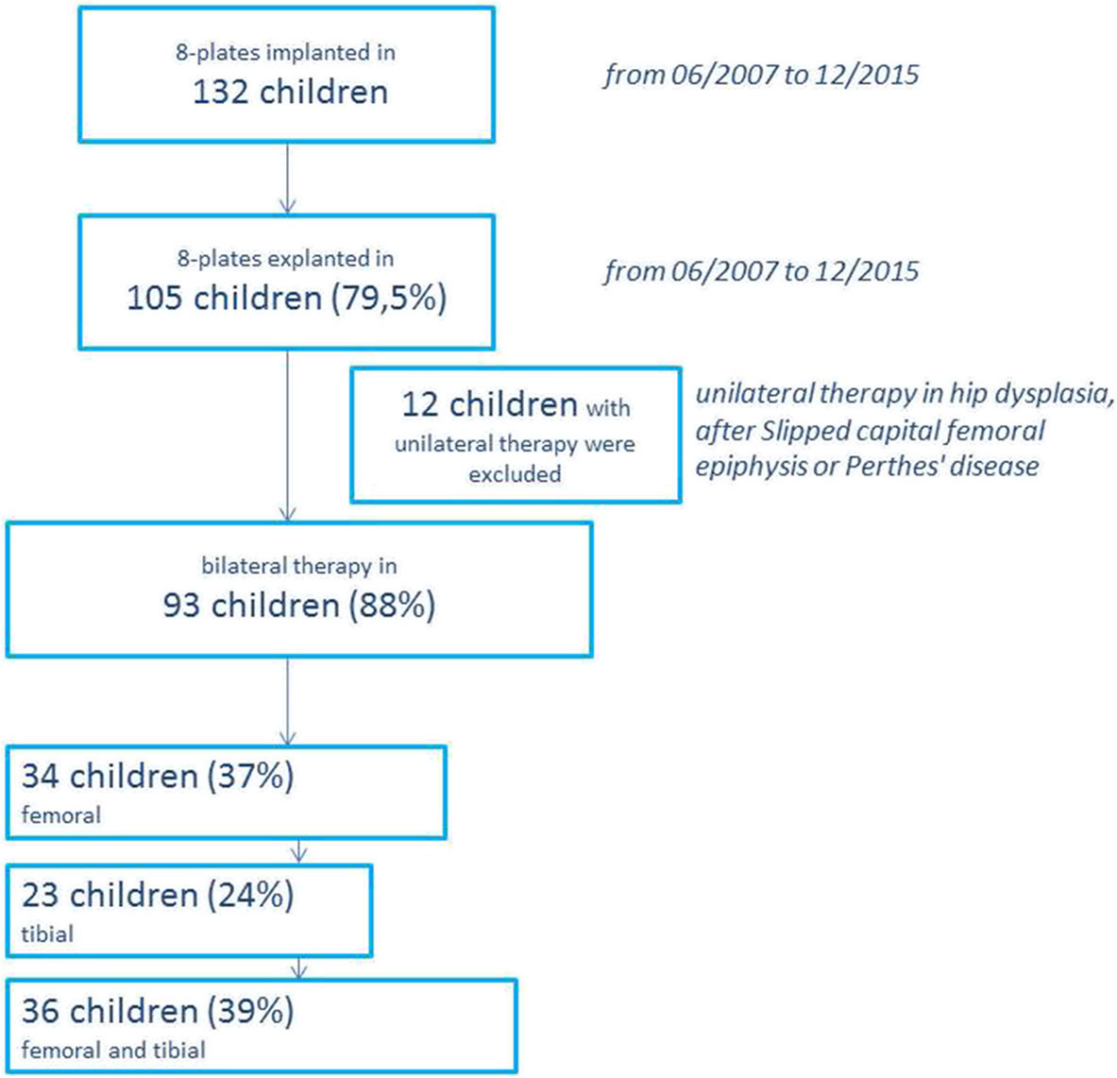
Flowchart showing the patient population included

Between July 2007 and July 2015, 198 eight-Plates were implanted near the knee in 132 children suffering from genu valgum to modulate growth. By 31 December 2015, they had been removed from 105 patients. Unilateral treatment was excluded. All follow-up examinations until 31 December 2016 were included in the analysis. The median follow-up period was therefore 46 months (12–102 months). (Fig. 2).

The median age at implantation was 12 (7–16) years. 45.7% of the patients (*n* = 48) were female. 43 children (41%) were obese at the time of implantation according to age- and gender-standardized BMI. Table 1 contains a list of secondary diseases. The median time from implantation to explantation of implants was 13 +/−6.44 months.

**Table 1:** Secondary diagnoses of treated children upon implantation: absolute and as percentage values in descending frequency

Additional diagnosis	Absolute number	Percentage
Obesity	43	40.9%
Chunky feet	6	5.7%
Syndromal disorders	6	5.7%
Noonan’s syndrome	1	1.0%
Trisomy 5	1	1.0%
Trisomy 21	4	3.8%
Scoliosis	5	4.8%
Slipped capital femoral epiphysis	5	4.8%
Congenital patellar dislocation	5	4.8%
Infantile cerebral palsy (ICP)	4	3.8%
Enchondromatosis	4	3.8%
Mental retardation	4	3.8%
Perthes’ disease	3	2.9%
Malignomas	3	2.9%
Epiphyseal dysplasia	3	2.9%
Schlatter’s disease	2	1.9%
Juvenile idiopathic arthritis	2	1.9%
Scheuermann’s disease	1	1.0%

**Fig. 3:**
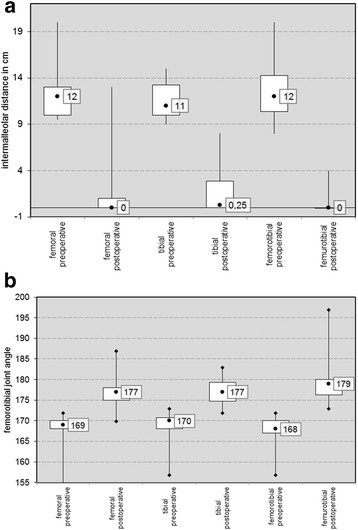
Graph (box plot) of the development of **a** the IMD and **b** the aFTA preoperatively and postoperatively as a function of localization

The change in joint angles (aFTA, aLDFA and mMPTA) and the intermalleolar distance (IMD) are shown in Table 2 as a function of the location treated. (Table 2) The development of the IMD and aFTA from implantation to explantation is shown in the graph in Fig. 3 as a function of location. Only the decrease in the IMD in the femorotibial group was statistically significant (*p* = 0.042); all other values improved, albeit insignificantly.

**Table 2:** Patients’ details (quantity, gender, age, obesity, time until explantation) and the development of joint angles (aFTA, aLDFA and mMPTA) and the intermalleolar distance (IMD) as well as relapses, overcorrections and incomplete corrections as a function of the area treated (bilateral femoral medial, bilateral tibial medial and bilateral femorotibial)

	Physiological value range	Femoral medial	Tibial medial	Tibial and Femoral medial
Number		34	23	36
Girls absolute / %		13 / 15%	7 / 30%	19 / 53%
Age at implantation median (min-max)		13 +/− 1.63 years	12 +/− 1.79 years	12 +/− 1.79 years
Time to explantation in months median (min-max)		14 +/− 5.05 months	17 +/− 5.46 months	13 +/− 7.33 months
Number of obesity absolute / %		18 / 53%	9 / 39%	11 / 30,6%
*Before surgery*				
anatomical femorotibial angle (aFTA) median (min-max)		169 +/− 2.76	170 +/− 2.43	168 +/−2.88
intermalleolar distance in cm median (min-max)		12 +/− 2.5	11 +/− 2.34	12 +/−3.04
anatomical lateral distal femoral angle (aLDFA) median (min-max)	81 (79–83)	*77 +/−2.01*	81 +/− 1.90	*76 +/−3.25*
medial proximal tibial angle (MPTA) median (min-max)	87 (85–90)	89 +/−0.98	*92,5 +/−2.82*	*91 +/−4.75*
*at material removal*				
anatomical femorotibial angle (aFTA) median (min-max)		177 +/−3.47	177 +/− 2.97	179 +/− 5.31
intermalleolar distance in cm median (min-max)		0 +/−2.85	0,25 +/− 2.37	0 +/−1.17
anatomical lateral distal femoral angle (aLDFA) median (min-max)	81 (79–83)	84.5 +/−4.25	82 +/− 2.01	84.5 +/−2.88
medial proximal tibial angle (MPTA) median (min-max)	87 (85–90)	89 +/− 1.5	88 +/−2.51	88 +/−3.27
Recurrence absolute/ %		1 / 2.9%	1 / 4.3%	2 / 5.5%
Overcorrection absolute/ %		1 / 2.9%	0	2 / 5.5%
Incomplete correction due to physiological growth gap closure absolute/ %		2 / 5.9%	2 / 8.6%	2 / 5.5%

Presented in the median (standard deviations) as well as absolute and percentage values

The evaluation of the axial correction as a function of age for implantation is shown in Table 3. It is found that the correction potential decreases with increasing age at implantation. Also the time to explantation was tendentially longer in older children at implantation. However, a large scatter without statistical significance was found.

**Table 3:** Patients’ details depending on age at implantation are shown for time until explantation and the development of joint angles aFTA and the intermalleolar distance (IMD)

	*Before surgery*		*at material removal*		
Age at implantation	anatomical femorotibial angle (aFTA) median	intermalleolar distance in cm median	anatomical femorotibial angle (aFTA) median	intermalleolar distance in cm median	Time to explantation in months median
7	169 +/− 2.76	15,5 +/− 2.45	187 +/− 2.89	0,5 +/−1.17	10 +/ 5.5
8	166,5 +/− 3.76	10 +/− 2.98	177,5 +/− 5.81	0 +/− 1.98	15 +/− 5.02
9	169 +/− 2.56	10,25 +/− 2.56	180 +/− 4.76	0 +/− 1.06	17 +/− 7.32
10	168+/− 2.65	12 +/− 2.67	177 +/− 3.78	0 +/− 1.89	11 +/− 5.87
11	168,5 +/− 1.98	12 +/− 2.10	179,5 +/− 3.56	0 +/− 2.01	14 +/− 5.7
12	169 +/− 2.79	11 +/− 2.65	177 +/− 3.45	0 +/− 2.32	17 +/−5.98
13	170 +/− 2.76	11 +/−2.57	177 +/− 4.11	0,5 +/− 1.56	11 +/−6.56
14	169 +/− 2.87	12 +/− 2.71	175 +/− 3.98	0,5 +/− 1.87	11 +/− 5.21
15	169 +/− 2.78	12 +/− 2.73	174 +/− 3.22	4 +/− 3.61	24 +/−4.6
16	169,5 +/− 2.67	11 +/− 3.06	174 +/− 3.42	1,75 +/− 2.21	13 +/−6.78

Presented in the median with standard deviations

**Fig. 4:**
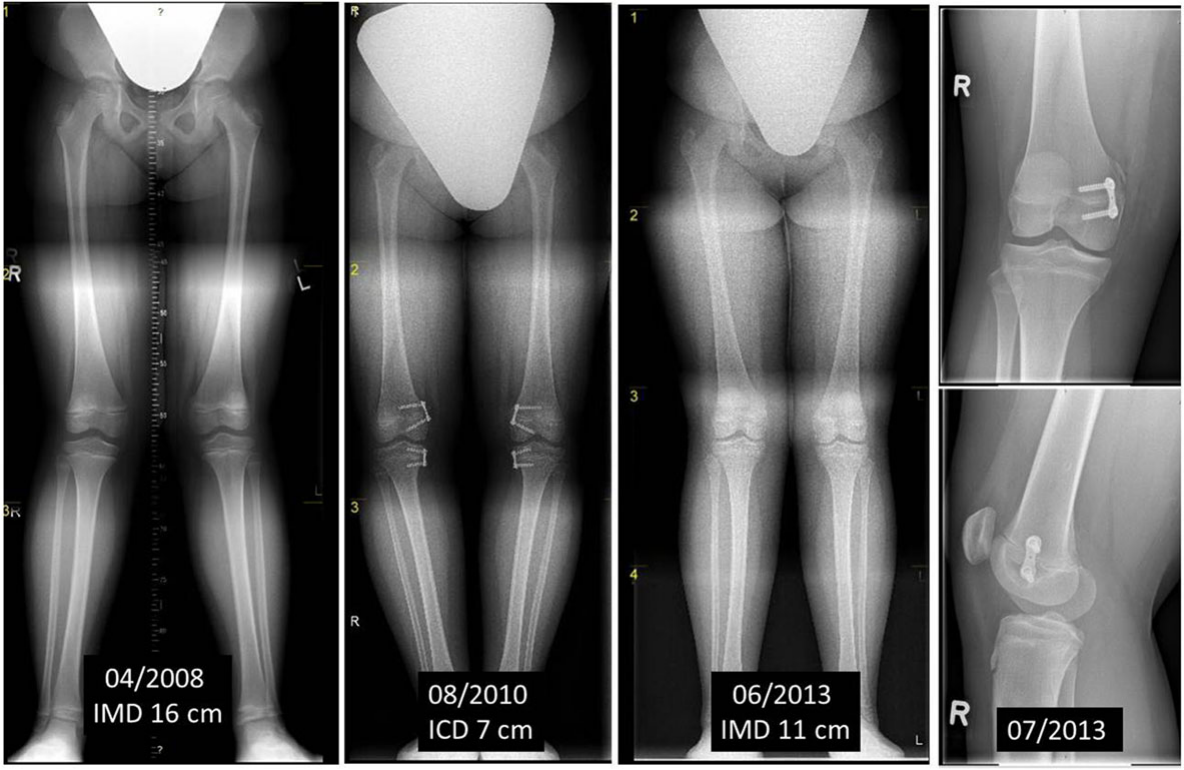
9-year-old adipose girl with genu valgum (IMD 16 cm) treated with femorotibial eight-Plates in April 2008 and growth enabled following material removal upon overcorrection (ICD 7 cm) in August 2010. Clinical relapse with IMD 11 cm in June 2013 with femoral error determined by X-ray and repeated hemiepiphysiodesis

In 4 children (3.8%), relapse occurred after correction and removal of the material. The median age of these children during implantation was 11 years (7–12 years). Hemiepiphysiodesis was repeated on 2 of these children aged 7 and 9 years (Fig. 4). The 2 others were not re-treated owing to the limited remaining potential growth. There were no significant differences between the location of treatment.

Wound healing problems occurred in 3 children (2.9%), but were cured in 2 of them by conservative treatment. In 1 boy, additional surgery was required due to suture granuloma. There were no significant differences between the areas treated here either.

In 3 cases, the overcorrection of genu valgum necessitated additional surgery. In these cases, the lack of compliance was the cause of overcorrection. A mutual implantation of 8- plates was impossible because of the physiologically terminated growth.

**Fig. 5:**
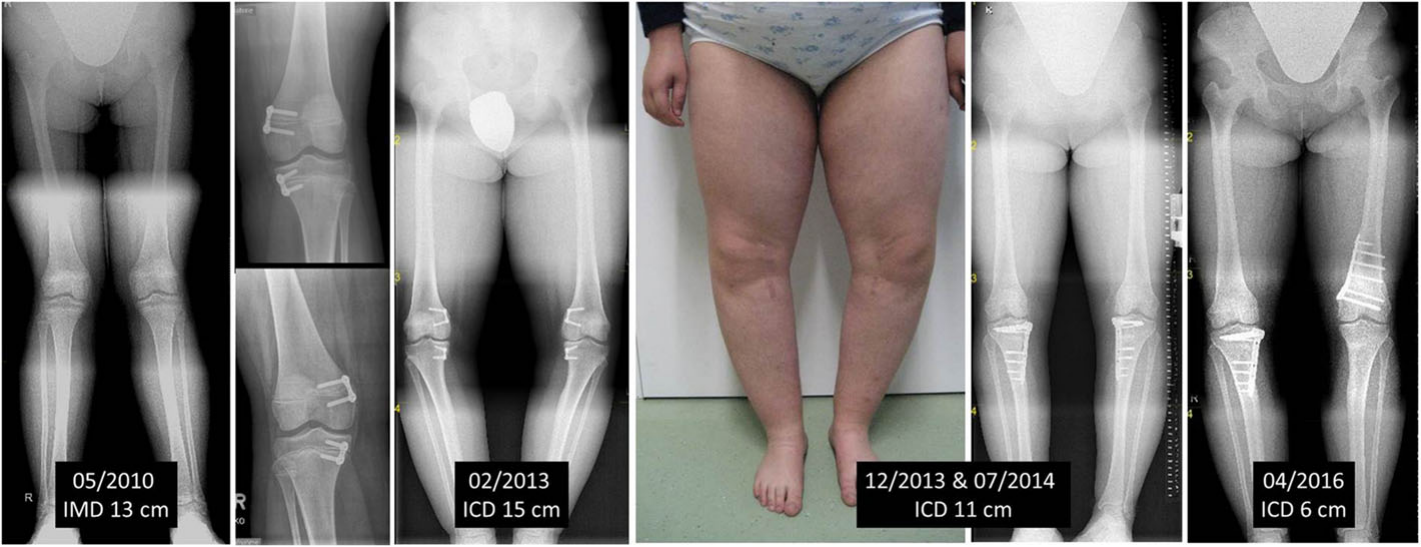
9-year-old adipose girl with genu valgum (IMD 13 cm) treated with femorotibial eight-Plates in May 2010 but no follow-up examination owing to a lack of compliance. Consultation in February 2013 with massive overcorrection (ICD 15 cm) and closed growth plates. Therefore, progressive bilateral tibial and right femoral corrective osteotomy has yet to be carried out

In 2 of these patients, corrective osteotomy had to be carried out owing to massive overcorrection (Fig. 5). In 1 patient, lateral eight-Plates were implanted. Once again, there were no significant differences between the areas treated.

In 1 case, when the material was removed, a screw broke off. However, this had no clinical relevance.

## Discussion

In our study, all patients with genu valgum responded well or even very well to growth modulation by means of temporary hemiepiphysiodesis through the epiphysis-bridging implantation of eight-Plates. Regardless of the position of hemiepiphysiodesis, the median IMD was corrected to 0 (0–13) cm and the anatomical femorotibial angle by an average of 9 (5–15)°. These results are comparable to the values reported in the literature for aFTA of 7–12° [17, 19]. In some studies, eight-Plates have been shown to have similar results to Blount staples and percutaneous epiphysiodesis using transphyseal screws (PETS) regarding the correction potential of the deformity [13–17, 19, 20]. As expected, in our analysis we found femorotibial treatment to have on the whole a higher corrective potential than simply tibial or femoral treatment, even though no significant differences regarding the area of treatment were observed in our study (Fig. 3). The assumption that this results in a higher potential for overcorrections or relapses was not confirmed with any significance in our study, despite the observation of a slight trend. (Table 2) This assumption has not been adequately proved using data from the literature either [16, 21, 22].

Regarding complication rates, studies of the use of Blount staples indicate more loosening and even damage to growth plates than when using eight-Plates. This is accounted for by the staples’ more rigid construction [19, 20]. Furthermore, Jelinek et al. reported that the implantation and explantation of eight-Plates is in each case about 10–12 min quicker than fitting and removing Blount staples [20]. However, both methods are considered to entail relatively few complications compared to PETS (percutaneous epiphysiodesis using transphyseal screws) [13, 23]. For example, corrective work is required in up to 18% of cases involving PETS [23]. In our examination, operational complications occurred in 2.8% of cases (1 wound infection, 3 corrective osteotomies following overcorrections). In the literature, complication rates (mainly breaking screws) of 6–16% are reported for eight-Plates [19, 24]. In our study, there was only a single case of a broken screw; furthermore, it did not have any clinical relevance after the material had been removed, enabling growth to resume. The high number of screw fractures reported in the literature may be caused by the convergence of screws when inserted or screws being overtightened (cold welding) – although there is no evidence of this happening in recent literature [24]. More complications are reported in the literature in connection with pathological physiology (dysplasia, Mb. Blount, etc.) than in children with normal physiology [19]. Relapses only occurred more frequently than usual in combination with previously treated club feet (3 out of 4, 75%). It is hence possible that a development disorder is associated with club feet and genu valgum, or that gait alteration owing to the treated club feet contributes to the development of axial deformities. Although there is no evidence for these hypotheses in larger cohorts, there are small studies in connection with genetic syndromes which may suggest this phenomenon [4].

Overcorrection after hemiepiphysiodesis is the subject of controversial debate. Due to the rebound phenomenon, a mild varus is especially recommended for children with a risk profile (obesity, epi. Dysplasia, etc.) and with still high growth potential in order to avoid a relapse as growth progresses [21, 22]. However, the line between this and pathological overcorrection is fluid and not defined in the literature [22]. In our study, corrections requiring surgery arose in 3 cases. In all cases, a key factor was the lack of patient compliance, for the families concerned failed to attend the regular follow-up examinations after implantation. Only when massive valgus deformities materialized did they consult the doctor. Such relapses are reported in the literature in particular depending on the patients’ age and underlying diseases, with rates in some cases of 100%. [3] Incomplete corrections also occurred in 6% of cases (*n* = 6), although only patients with low growth potential and an average implantation age of 14 years were affected. No link was found with the area of implantation. Faster leg straightening with Blount staples is postulated owing to more rigid fixation with compression of the growth plate, especially in patients with only a small potential for growth [16, 17]. However, this could not be conclusively confirmed in further studies [19, 20].

## Conclusion

Growth modulation by means of temporary hemiepiphysiodesis using eight-Plates to treat genu valgum is a gentle and effective procedure. Particularly in young patients with high growth potential and risk groups such as obese patients, slight overcorrection is desirable due to the rebound phenomenon. In children with only low growth potential (older than 14 years), owing to the low correction potential, the indication should be strictly reviewed and the possible failure of therapy should be discussed with the patient. In the literature, the use of Blount staples is sometimes recommended for such cases. No differences were observed regarding the positioning of the plates. Patient compliance (especially regarding regular follow-up examinations) is essential if complications are to be avoided.

## Limitations

The main limitation of our study is its retrospective design with no comparison group. Moreover, it is an inhomogeneous group with a fluctuating follow-up period, and explantation has not yet been performed on all patients.

## Abbreviations

aFTA: Anatomical femorotibial angle
aLDFA: Anatomical lateral distal femoral angle
K-wires: Kirschner wires
MD: intermalleolar distance
MPTA: Medial proximal tibial angle
